# Enhancing Bioproducts in Seaweeds via Sustainable Aquaculture: Antioxidant and Sun-Protection Compounds

**DOI:** 10.3390/md20120767

**Published:** 2022-12-07

**Authors:** Doron Yehoshua Ashkenazi, Félix L. Figueroa, Nathalie Korbee, Marta García-Sánchez, Julia Vega, Shoshana Ben-Valid, Guy Paz, Eitan Salomon, Álvaro Israel, Avigdor Abelson

**Affiliations:** 1 School of Zoology, Tel Aviv University, Ramat Aviv, Tel Aviv-Yafo 69978, Israel; 2 Israel Oceanographic & Limnological Research Ltd. (PBC), Tel Shikmona, P.O. Box 9753, Haifa 3109701, Israel; 3 Andalusian Institute for Biotechnology and Blue Development (IBYDA), Experimental Center Grice Hutchinson, Malaga University, Lomas de San Julián, 2, 29004 Málaga, Spain; 4 Israel Oceanographic and Limnological Research, National Center for Mariculture, P.O. Box 1212, Eilat 8811201, Israel

**Keywords:** seaweeds, integrated aquaculture, mycosporine-like amino acids, phenolic compounds, pigments, antioxidants, sun protection factor, bioproducts, *Ulva*, *Gracilaria*

## Abstract

Marine macroalgae are considered an untapped source of healthy natural metabolites and their market demand is rapidly increasing. Intertidal macroalgae present chemical defense mechanisms that enable them to thrive under changing environmental conditions. These intracellular chemicals include compounds that can be used for human benefit. The aim of this study was to test cultivation protocols that direct seaweed metabolic responses to enhance the production of target antioxidant and photoprotective biomaterials. We present an original integrated multi-trophic aquaculture (IMTA) design, based on a two-phase cultivation plan, in which three seaweed species were initially fed by fish effluents, and subsequently exposed to various abiotic stresses, namely, high irradiance, nutrient starvation, and high salinity. The combined effect of the IMTA’s high nutrient concentrations and/or followed by the abiotic stressors enhanced the seaweeds’ content of mycosporine-like amino acids (MAAs) by 2.3-fold, phenolic compounds by 1.4-fold, and their antioxidant capacity by 1.8-fold. The Sun Protection Factor (SPF) rose by 2.7-fold, and the chlorophyll and phycobiliprotein synthesis was stimulated dramatically by an order of magnitude. Our integrated cultivation system design offers a sustainable approach, with the potential to be adopted by emerging industries for food and health applications.

## 1. Introduction

In an era of climate change, marine macroalgae (seaweeds) have the potential to play a crucial role in food security coping with the world’s uprising challenges [1]. There are several advantages to producing seaweed biomass. Compared to land aquaculture, seaweed aquaculture does not require excessive arable land, freshwater, or large amounts of fertilizers or pesticides [2,3]. Moreover, seaweed cultivation provides many ecosystem services [4]. As primary producers, and as the most productive marine macrophytes on a global scale, seaweeds fix carbon dioxide and produce life-supporting oxygen [5,6,7], attributes that may help to mitigate and reduce greenhouse gases in the efforts to counter global warming [8,9,10]. Seaweeds are also extractive species, specialized in assimilating dissolved nutrients and pollutants, cleaning the natural aquatic environment and, thereby, contributing to maintaining the overall ecological balance in coastal ecosystems [11,12,13]. Globally, seaweed aquaculture has tripled over the last few decades, reaching 32.4 million tonnes of fresh biomass in 2018, with an estimated market value of US $13.3 billion [14]. Seaweed products are used in major industries, such as food, animal feed, textiles, pharmaceuticals, medicine, and cosmetics [15,16,17,18]. However, their true potential is far from being fully utilized [19].

Integrated multi-trophic aquaculture (IMTA) is a sustainable, ecologically sound approach that advocates the integration of fed species, such as finfish, with inorganic and organic extractive species from lower trophic levels (e.g., seaweeds and shellfish). Fed finfish monocultures may generate surplus discharge of organic matter and dissolved nutrients. The nutrient-rich fish effluents can negatively affect natural ecosystems, causing habitat modification, water quality degradation, and coastal eutrophication. The seaweeds in the IMTA system assimilate the fish waste, which is rich in dissolved ammonia, phosphate, and carbon, to form new biomass and synthesize needed natural materials. In doing so, the seaweeds are able to clean and treat the water, minimizing negative environmental impacts and improving the economic viability, while increasing the quality of the cultivated marine crop [20,21]. In a previous study we addressed the high biofiltration abilities of seaweeds, evaluating and maximizing their performance as part of an IMTA system [22]. We demonstrated a nutrient removal efficiency of up to 100% together with an outstanding nutrient removal rate of 4 g total ammonia nitrogen (TAN) m^−2^ day^−1^. Recently [23], we have focused on increasing the concentrations of primary metabolites in seaweeds, such as proteins, functional carbohydrates, and important minerals for human nutrition and health. The current work focuses on metabolic stimulation of seaweed secondary metabolites and bioproducts with potential health benefits for human society.

The Levant basin in the eastern Mediterranean Sea is a particularly stressful environment, experiencing oligotrophic conditions and high average temperatures, together with fluctuating abiotic conditions on a daily basis [24,25,26]. To thrive under such conditions, seaweeds have adapted an arsenal of unique natural chemical defenses that are not found in other organisms, such as phenolic compounds, distinctive protective pigments, and natural sun-screening substances, such as mycosporine-like amino acids (MAAs) [27,28,29,30]. Seaweed secondary metabolites inherently possess strong bioactive attributes, and have been shown to exhibit antioxidant, anti-viral, anti-biotic, anti-cancer, anti-fungal, anti-diabetic, and photoprotective properties, with a range of biological activities proven to have a positive effect against chronic diseases in humans [18,28,31,32,33].

MAAs are strong natural photoprotectors largely found in red seaweeds [34,35]. They are ultraviolet radiation (UVR)-absorbing compounds characterized by high absorption in the 310–362 nm wavelength range, and are considered to have evolved as a natural defense against chronic UVR exposure in sunlight-rich, shallow-water habitats. MAAs protect the living cell by absorbing the harmful sun radiation, dissipating its energy as heat and, consequently, minimizing the production of reactive oxygen species (ROS) [36,37]. MAAs are considered ‘multipurpose’ secondary metabolites, since they also present strong antioxidant, anti-aging, and anti-inflammatory properties [35,38]. In humans, exposure to UVR can be harmful to the skin, inducing photo-aging, loss of skin resilience, formation of wrinkles, and even skin cancer. MAAs can be used to produce environmentally friendly ultraviolet (UV) filters that will present a natural alternative to the common commercial synthetic filters [39]. Natural pigments, such as chlorophylls and phycobiliproteins (phycoerythrin, phycocyanin), have attracted special attention in the fields of nutrition, cosmetics, and pharmacology, as they have been found to display various beneficial biological activities [40]. Phenolic compounds are also secondary metabolites, characterized as antioxidant and stress-protective compounds against biotic and abiotic stressors, such as grazing and fouling organisms, excessive UVR, and metal contamination [41,42]. 

Despite their biotechnological potential, to date, seaweed biomass has been used primarily as raw material for food and for hydrocolloid industries (agar, alginate, and carrageenan), with less than 1% of the global seaweed production used for high-value, health-promoting products [17,43]. Currently, however, following the public’s increasing pursuit of a healthy lifestyle and a consumer preference for quality food and natural products, there is growing demand for seaweed products [44,45]. The leap from raw commoditized seaweed biomass to functional biomass has not yet occurred, where one reason is the lack of appropriate technologies. The future challenges of the seaweed aquaculture industry will, therefore, require the development of new cultivation methods dedicated to ensuring a sustainable supply of seaweed biomass with consistently high levels of targeted bioactive compounds [43,46,47]. 

The seaweeds’ intracellular chemical composition and, thus, their metabolite levels are regulated by their surrounding environmental conditions, the majority of which relate to irradiance, light quality [31,48,49,50], salinity, and the availability of nutrients. Additional factors include biological pressures, such as grazing and allelopathy, life cycle stages, geographic location, seasonality, and more [24,51,52,53,54,55].

The present study was aimed at increasing target secondary metabolites in local model seaweeds by altering their growth conditions, namely, nutrient depletion, excessive radiation, and high salinity. We evaluated the levels of MAAs, chlorophylls, phycobiliproteins and polyphenols, together with the seaweeds’ antioxidant capacity and Sun Protection Factor (SPF) capabilities. We operated a novel IMTA cultivation design based on a two-phase cultivation scheme, using finfish and three intertidal seaweed species, *Ulva rigida*, *Gracilaria conferta*, and *Hypnea musciformis*, known for their high productivity and special bioactive attributes [18,56,57,58]. Our overall goal was to develop a well-defined methodology to produce an enhanced functional seaweed biomass, and to establish a multidisciplinary and accessible cultivation approach for the rapidly evolving seaweed aquaculture and health-promoting industries.

## 2. Results

Supplementary S2.1 presents the nutrient levels measured in the integrated system. The experimental design and system layout is depicted in Figure 6 in the Materials and Methods section, presenting the two-phases approach cultivation scheme, including initial integrated cultivation alongside fish culture, following a second phase, exposing the seaweeds to abiotic environmental stress conditions. The statistical information for the different tests is summarized in Supplementary S1. When the results of more than one test are given, they are referred to Supplementary S1. The general scheme depicting how each treatment affects the seaweeds, leading to a specific metabolic optimization, is summarized in Table 3.

### 2.1. Mycosporine-like Amino Acids (MAAs)

**Figure 1:**
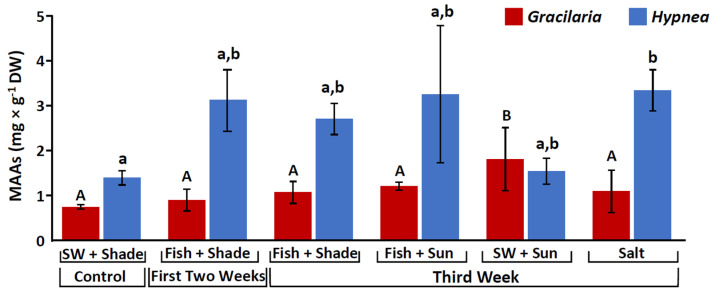
Mycosporine-like Amino Acids (MAAs) content (mg g^−1^ DW), for *Gracilaria conferta* (red) and *Hypnea musciformis* (blue), cultivated under the different culture conditions. SW (seawater). Statistical analysis was performed for each species separately. Different letters indicate significant differences (uppercase letters: *Gracilaria*, lowercase: *Hypnea*).

In the current work, MAAs were only identified in red algae. Both *Hypnea musciformis* and *Gracilaria confera* showed significant differences in their MAA content among the different treatments (One-way ANOVA, *F*_5,14_ = 4.8, 3.04, *p* = 0.009, 0.046, respectively, Supplementary S1, Table 1, Figure 1). *H. musciformis* presented the highest MAA levels when integrated with fish, and under high salinity, while *G. conferta* showed similar values between treatments, with some increase when exposed to full sunlight. Generally, both red seaweed species contained two main MAAs: palythinol and shinorine. *G. confera* also exhibited relatively high levels of porphyra-334, low quantities of palythine (0.014–0.2 mg g^−1^ DW), and trace level of asterina-330. *H. musciformis* additionally contained palythine as a main MAA and had small amounts of asterina-330 (0.01–0.05 mg g^−1^ DW), while porphyra-334 was present in *H. musciformis* in only few samples (the content of each individual MAA is summarized in Supplementary S2.2). 

**Figure 2:**
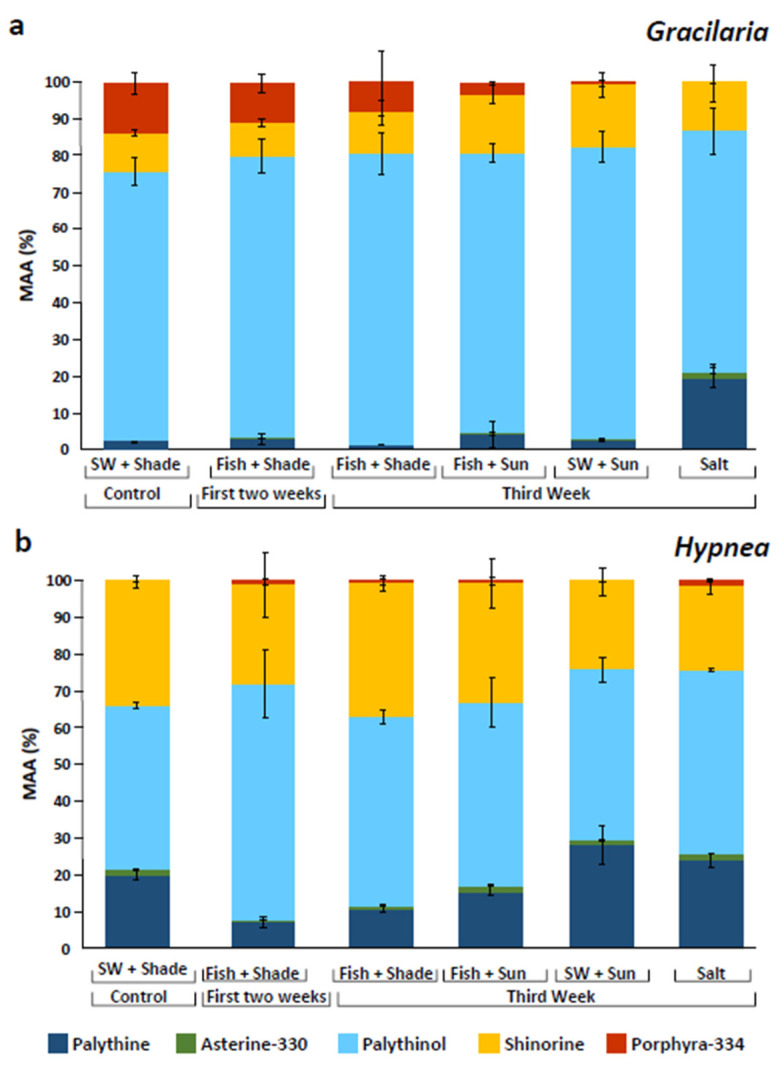
MAA proportions (%) for *Gracilaria conferta* (**a**) and *Hypnea musciformis* (**b**), cultivated under different culture conditions. SW (seawater).

Overall, the MAA composition was affected by the different treatments (Figure 2). Palythinol fluctuated between treatments and ranged between 66 and 80% for *G. conferta*, and between 45 and 64% for *H. musciformis*. Shinorine had the highest percentage in *G. confera* under the seawater and full sunlight treatment with up to 17%, and the highest percentage in *H. musciformis* when integrated with the fish (36%). Palythine showed significant alternation among treatments and exhibited the highest percentage in *G. conferta* under the high salinity treatment (20%), and in *H. musciformis*, palythine showed significantly higher levels under treatments that received only seawater supply (up to 28%). Porphyra-334 appeared in relatively high levels in *G. conferta* following the initial two weeks and showed a significant decrease after the third week. Overall, *H. musciformis* displayed a significantly higher MAA concentration compared to *G. conferta* (permutation ANOVA, *F*_1,38_ = 33.4, *p* < 0.0001). 

### 2.2. Pigment Content (Chlorophylls and Phycobiliproteins)

Chlorophyll *a* and *b* were evaluated for *Ulva rigida*, and chlorophyll *a* and *d* for the Rhodophyta species, *Gracilaria conferta* and *Hypnea musciformis* (Table 2). For all species, significant differences were observed among treatments, both for chlorophyll *a* and for the total chlorophyll contents (*p* < 0.0001, Table 2, Supplementary S1). Overall, two distinct patterns were observed; firstly, seaweeds grown in the integrated cultures had significantly higher chlorophyll levels compared to the seaweeds cultivated in treatments with only seawater supply (Supplementary S1, Table 2). Additionally, treatments that received 100% sunlight (Seawater + Sun, high salinity) presented the lowest chlorophyll levels. The maximal chlorophyll *a* difference between treatments was up to 1250, 522, and 393% for *U. rigida*, *G. conferta*, and *H. musciformis*, respectively. Similar differences were observed for the total chlorophyll levels. Secondly, chlorophyll levels (*a*, *b*, *d* and total chlorophyll) accumulated extensively following the initial two weeks, while showing a significant decrease after the third week (Table 2). Differences between the second and third weeks of cultivation ranged up to 234, 140, and 170% for *U. rigida*, *G. conferta*, and *H. musciformis*, respectively (calculated for the Fish + shade treatment, chlorophyll *a*, Table 2). For all treatments, *U. rigida* presented the highest total chlorophylls levels, followed by *H. musciformis* and *G. conferta* (Permutation ANOVA, *F*_2,51_ = 14.62, *p* < 0.0001, Table 2). Phycoerythrin and Phycocyanin were evaluated for *H. musciformis* and *G. conferta.* Overall, the patterns observed for the chlorophylls, including higher concentration in the integrated tanks and higher levels following the second week, appeared in a similar way for the red pigments (Table 2). The maximal phycoerythrin difference between treatments ranged up to 1150 and 714% for *G. conferta* and *H. musciformis*, respectively, and the phycoerythrin difference ranged up to 815 and 1105%, respectively. Overall, *H. musciformis* presented higher phycobilins levels compared to *G. conferta* (Permutation ANOVA, Supplementary S1, Table 1 and Table 2).

### 2.3. Antioxidant Activity 

**Figure 3:**
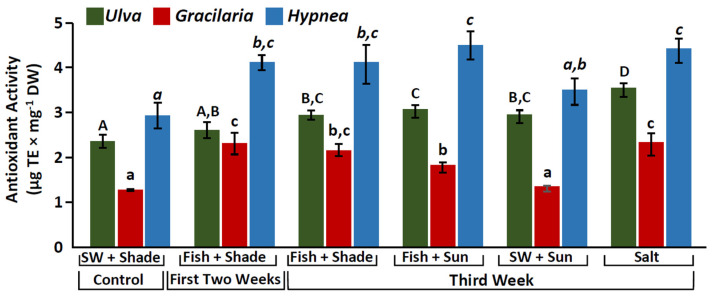
Antioxidant activity (µg TE mg^−1^ DW) for *Ulva rigida* (green), *Gracilaria conferta* (red), and *Hypnea musciformis* (blue), cultivated under the different culture conditions. SW (seawater). Statistical analysis was performed for each species separately. Different letters indicate significant differences (uppercase letters: *Ulva*, lowercase: *Gracilaria*, italics: *Hypnea*).

Significant differences were observed among treatments (One-way Anova, *p* < 0.0001, Supplementary S1, Figure 3). A consistent pattern was observed, where in most cases, seaweeds grown in the integrated cultures had a significantly higher antioxidant capacity compared to the control seaweeds that were cultivated with only seawater supply (*p* < 0.008, Supplementary S1, Figure 3). Those differences were considerable, up to 150–180% (Figure 3). Integrated seaweed cultures usually presented similar antioxidant levels, especially for *Hypnea musciformis* (*p* > 0.08, Supplementary S1, Figure 3). Seaweeds cultivated under the high salinity treatment presented the highest antioxidant values (Figure 3). Overall, *H. musciformis* presented the highest antioxidant capacity, followed by *Ulva rigida* and *Gracilaria conferta* (One-way ANOVA, *F*_2,57_ = 88.2, *p* < 0.001, Table 1).

### 2.4. Phenolic Compounds

**Figure 4:**
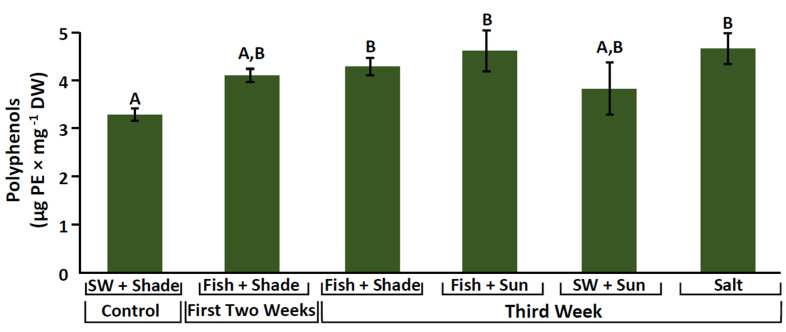
Phenolic content (μg PE mg^−1^ DW) for *Ulva rigida* cultivated under the different culture conditions. SW (seawater). Different letters indicate significant differences.

Phenolic compound content was evaluated for *Ulva rigida*. The seawater control treatment showed, in most cases, a significantly lower phenolic content compared to the other culture conditions (permutation ANOVA, *F*_5,15_ = 7.7, *p* < 0.001). Differences ranged up to 1.4 times higher. Other treatments showed only minor differences (Figure 4, Supplementary S1). The highest values of phenolic compounds were observed in the high salinity and the Fish + 100% sunlight treatments (reaching almost 5 μg PE mg^−1^ DW).

### 2.5. Sun Protection Factor (SPF)

**Figure 5:**
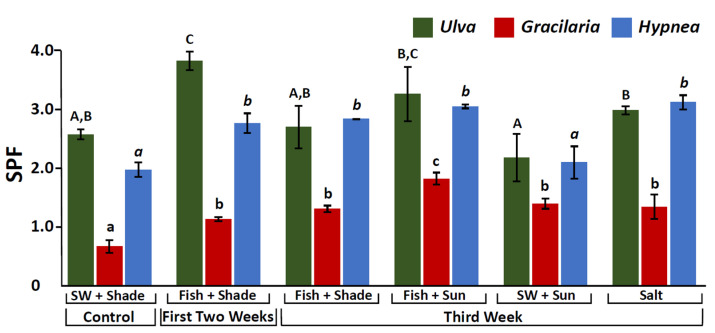
Sun Protection Factor (SPF) for *Ulva rigida* (green), *Gracilaria conferta* (red) and *Hypnea musciformis* (blue), cultivated under the different culture conditions. SW (seawater). Statistical analysis was performed for each species separately. Different letters indicate significant differences (uppercase: *Ulva*, lowercase: *Gracilaria*, italics: *Hypnea*).

For all the seaweed species, significant differences were found between the different culture conditions (One-way ANOVA, *p* < 0.001, Supplementary S1, Figure 5). SPF differences between treatments reached up to 160–270%. The seawater control (Seawater + Shade) usually presented the lowest SPF values. *Ulva rigida* and *Hypnea musciformis* had the highest SPFs in the integrated cultures and under the high salinity treatment, while *Gracilaria conferta* exhibited the highest SPF under the fish and 100% sunlight treatments (Figure 5).

## 3. Discussion

### 3.1. Stimulation of Mycosporine-like Amino Acids (MAAs) 

As MAAs are nitrogenous compounds with natural UVR screening and antioxidant properties, their levels are innately influenced by ammonium availability, irradiance, and oxidative stress conditions [35,59,60,61]. In the present study, the high availability of those factors not only increased the seaweed total MAA levels, but also altered their composition. In previous work, a high ammonium supply was shown to reduce the photoinhibition caused by high irradiance in the red macroalga *Grateloupia lanceola* [62]. 

Previous studies have examined how environmental abiotic conditions affect MAAs. Not only can seaweed MAA levels vary between environmental conditions, species, cultivation system (and additional factors), but their specific accumulation can be very flexible [31,61,63]. Thus, we believe that the assessment of additional species under novel IMTA schemes, based on data from previous studies, offers an important tool with which to establish an applicative method for a consistent seaweed-derived MAA production. 

MAA biosynthesis is mostly considered to be derived from conversion of the shikimate pathway, known for the synthesis of aromatic amino acids [34,64]. As presented in the current work, the coupling of high solar irradiance to generate sufficient energy for photosynthesis, together with the high availability of the required building blocks, N- and C-compounds [54], presents a particularly favorable environment for MAA production. Those conditions are largely met by integrated cultivation under an IMTA system. While the advantage of IMTA cultivation lies in supporting high MAA production in seaweed biomass, which has been demonstrated in previous studies [29,54,58,65], other studies have demonstrated a higher accumulation of MAAs under a low nutrient environment rather than a high one. This was explained by higher UVR penetration in the thallus, and by the competition with other processes for the use of nutrients under a low nutrient supply in oligotrophic waters [57]. MAA synthesis is also largely dependent on the irradiance quality and quantity. Korbee et al. [50] showed that in *Porphyra leucosticte*, MAA synthesis was stimulated by blue light and a high ammonium concentration. Karsten and Wiencke [63] demonstrated that intertidal seaweeds that were exposed to high solar radiation accumulated larger amounts of MAAs than subtidal seaweeds that received less light. Similar to the current work, Karsten et al. [66] showed that the concentrations of the MAAs shinorine, palythinol, and palythine increased considerably in the red seaweed *Chondrus crispus* during one-week exposure to a photosynthetically active radiation (PAR) of 400–700 nm. They also found that shinorine accumulation was more affected by UVR, while palythinol and palythine accumulation was more affected by PAR. The effect of UV-A, UV-B, and PAR on different MAA concentrations was also demonstrated by Peinado et al. [61], suggesting that different light regimes may induce different MAA profiles.

The seaweed total MAA content in the present study was in line with those of other species in previous works (1.8 and 3.3 mg g^−1^ DW for *Gracilaria conferta* and *Hypnea musciformis,* respectively). To the best of our knowledge, there is little published work on *H. musciformis* and MAAs. *Gracilaria* species can yield MAA concentrations that range between 1.75 to 2.4 mg g^−1^ DW [57,58,59], while in other Rhodophyta species, such as *Porphyra* spp., MAA contents may range from 5 to 10 mg g^−1^ DW [50,67,68]. Unlike previous studies, in the present research, palythinol was the major dominant MAA for both *G. conferta* and *H. musciformis*, reaching up to 80% of the total MAA content. Among the MAAs identified, palythine was previously demonstrated to present the highest antioxidant capacity [38]. Lawrenze et al. [69] showed that palythine extracted from the red macroalga *Chondrus yendoi*, in addition to its antioxidant capacity (anti-photoaging substance), may present photoprotection attributes against a wide range of adverse effects in HaCaT keratinocytes exposed to solar-simulating and UV-A radiation, i.e., protection against two types of DNA photolesions: cyclobutane pyrimidine dimers and 8-oxo-7,8-dihydroguanine. In the current work palythine levels were at their peak during exposure to high irradiance and salinity, which may indicate that the seaweeds used palythine as a protective antioxidant agent and as a physiological response to stress. Thus, palythine has been shown to be an extremely effective multifunctional photoprotective molecule, with the potential to be developed as a natural and biocompatible alternative to currently approved UVR filters [69].

Generally, a high salinity environment presents a stressful setting that may also facilitate the accumulation of MAAs in seaweeds. In marine algae, MAAs were suggested to have an osmotic function that evolved to cope with high salinity conditions. It was observed that in hypersaline water, certain marine algae may synthesis MAAs in high concentrations in order to reduce their cellular salt concentrations and restore their osmotic balance [70,71]. Similarly, in the current study, *H. musciformis* exhibited an increased concentration of MAAs when exposed to high salinity and solar irradiance stress, presumably using the MAAs as an osmoregulatory agent. In the present work, we cultivated two red seaweed species and demonstrated that different species may present different responses to similar environmental conditions by acquiring different MAA profiles. This may play an important role in future applications, where there may be a biotechnological interest, for example, in producing a specific MAA for a specific use. Overall, because MAAs offer tremendous biotechnological potential for use as multi-purpose natural products in the health and cosmetic industries [35,39], there is great interest in developing a consistent method by which to optimize their production.

### 3.2. Enhancement of Pigment Content

The main light-harvesting pigments of seaweeds are chlorophylls, carotenoids, and various groups of additional accessory pigments specific to algal phyla, such as phycobilins in the Rhodophyta [46]. In the current work, the seaweeds’ pigment concentrations were naturally influenced by two main factors: irradiance and nutrient availability.

Pigment concentrations respond to ambient light levels and are linked to the algal productivity and energy metabolism, resulting in high-light or low-light acclimated seaweed tissue [53]. Algal physiological responses to light conditions include control over pigment levels and ratios. Classic works on the impact of light on seaweed pigmentation include those reported by Ramus et al. [72] and Beer and Levy [73], who documented pigment changes in *Ulva* sp. and *Gracilaria* sp. according to water depth and light intensity. Generally, pigment synthesis in seaweeds will increase with greater depth and when transferred to a deeper position in the water column (low irradiance), as a way to increase their effective light-harvesting cross-section, for efficient light-energy harvesting. This acclimation process particularly occurs in intertidal seaweeds, such as *Ulva*, *Gracilaria*, and *Hypnea*, which are exposed to light irradiation alterations in their natural habitat on a regular basis, due to the constant tidal changes of water depth. Figueroa and Niell [74] further described how different qualities of a continuous light (blue, green, or red light), can control and increase chlorophyll and phycobilin levels as adaptative responses to the underwater light environment. This process is also defined as chromatic adaptation. As nitrogenous compounds, phycobiliproteins and chlorophylls are also affected by N-availability. Under nitrogen-rich environments seaweeds may accumulate amino acids, proteins, and N-containing pigments, such as chlorophylls and phycobiliproteins [75,76]. In contrast, nitrogen-deprived conditions will result in reduced chlorophyll and soluble protein levels, such as RUBISCO [77].

This pigment acclimation process could be clearly observed in the current study through the significant differences seen between a nutrient-poor environment (the control/Seawater + Shade and Seawater + Sun treatments) and a nutrient-rich environment (seaweeds that received fish effluent) (Table 2). Under fish-integrated conditions, the seaweeds’ chlorophyll levels rose by up to 6-fold, phycoerythrin levels by up to 7-fold, and phycocyanin levels by up to 11-fold. Thus, the optimal growing conditions for enhanced pigment concentration, according to our findings, are those of integrated, nutrient-rich conditions, which provide and support sufficient energy and building blocks for pigment synthesis. Interestingly, however, under the same ideal environment (Fish + Shade, Fish + Sun), there was a dramatic decline (>50%) in all types of pigments and in all three seaweed species during the transition from the second to third week of the experiment. A similar phenomenon was described previously by Korbee-Peinado et al. [61], who demonstrated a continuous decrease in chlorophyll and phycobilin concentrations after 6 days of experimentation. Chromatic adaptation in algae may be relatively rapid. Under different culture conditions, chlorophyll and phycobiliprotein levels in seaweeds have been demonstrated to change several-fold within a matter of hours [74]. In the current work, a two-week cultivation period seemed to be more than sufficient to produce seaweeds with a high pigment content. It is possible that, given the high levels of resources (light and nutrients), the seaweeds tunnel the energy to pigment synthesis to support accelerated growth and productivity. Once this goal is achieved and the pigment concentrations reach saturation, the seaweeds may lower their pigment content to a constant ideal concentration in order to conserve energy, maintain growth, and adapt to their new environment. We conclude that in order to achieve high pigment levels for biotechnological needs, the seaweeds should be harvested at an early culture stage, ranging from hours and a few days to a few weeks after providing the required culture treatment.

Generally, chlorophyll and phycobiliprotein values were in line with the same and other edible seaweed genera in the literature, and also when compared to studies that had used advanced analytical extraction methods [30,78,79,80]. 

Natural pigments have attracted attention by the food and beverages industries, as well as by the animal feed, cosmetics, and pharmaceutical markets. Seaweed pigments have exhibited several positive bioactive activities, such as antioxidant and radical scavenging activities, and anti-inflammatory, anti-diabetic, and anti-cancer (several tumor types) properties [53,81,82,83]. Recently, chlorophylls have also been suggested for use as a potential therapeutic agent for treating COVID-19 [84,85]. Additionally, seaweed pigments can be used as artificial dyes/colorants [80]. The food colorant market is growing rapidly, estimated at 3.75 billion USD in 2022, and revealing a distinct consumer interest in natural food colors (especially those that exhibit health benefits) and in food additives that are non-synthetic and safer to consume [82]. Finding a consistent method for enhancing pigment levels in seaweeds, which can be mass-produced, may, therefore, present an important step in integrating seaweeds into the emerging food and pharma industries.

### 3.3. Increase in Antioxidant Activity and Phenolic Compounds

Intertidal seaweeds live in harsh environments where they are subjected to various abiotic and oxidative stress conditions that produce reactive oxygen species (ROS), which may lead to oxidative damage [60,86]. Seaweeds respond to these conditions by increasing an array of antioxidant defenses, which include ROS-scavenging enzymes and/or nonenzymatic antioxidative substances, and secondary metabolites, such as phenolic compounds, ascorbic acid, tocopherols, carotenoids, phycobiliproteins phospholipids, chlorophyll-related compounds, catechins, MAAs, polysaccharides, and more [53,87,88]. In the current study, the highest antioxidant capacity was observed under the high salinity conditions, and, additionally, in the integrated seaweeds that received a consistent, rich nutrient supply, provided by the fish effluents. Nutrient availability may have an impact on the growth and functioning of seaweeds in extreme environments [89]. Elevated nitrogen levels may have a key role in enabling the seaweeds to increase their antioxidant defenses against abiotic stressors, such as high solar and UV radiation [29,68,90,91]. This has been specifically described in Gracilariaceae and for *Gracilaria conferta* [57,92]. Huovinen et al. [62] also discussed the importance of ammonium in protection against high irradiance and in the recovery of algal photosynthetic activity. In this context, Lesser et al. [90] demonstrated that N-supplied algae were more resilient to high UVR compared to N-limited algae. The excess nitrogen aided the algal cell to repair UV-B-induced damage by increasing the turnover of critical proteins and protein–pigment complexes associated with photosynthesis. Thus, it can be safely assumed that seaweed cultivation via IMTA not only leads to rapid seaweed growth, but additionally creates a supportive environment that provides resources for seaweeds to protect themselves against stress and oxidation conditions, and eventually also boosts their chemical defenses. Interestingly, in the current work, there were no significant differences in the antioxidant capacity between the shaded cultures and those that were exposed to 100% sunlight, in which a higher antioxidant capacity would be expected. We presume that this could have been the result of self-shading and interactive photoprotection by the seaweeds’ thalli, which accumulated rapidly due to the optimal combination of culture season (spring bloom) and IMTA conditions [23]. As noted, high salinity was the additional factor that presented the highest antioxidant capacity for all the study’s three seaweed species—*Ulva rigida*, *Gracilaria conferta*, and *Hypnea muschformis.* Salinity plays a vital role in restricting the growth and development of seaweeds in the intertidal zones and estuaries [93,94]. Salinity stress may lead to rapid accumulation of ROS in seaweeds, thus leading to oxidative stress [95,96]. In the present work, it was evident that the three seaweed species had contended with high salinity by elevating their antioxidant capacity. It was previously documented that intertidal seaweeds have developed a strong ability to resist salinity changes [93,97]. *Ulva fasciata* and *Ulva prolifera*, specifically, have been reported to increase their antioxidant levels and antioxidant enzyme activities when exposed to stressful salinity environments [86,98,99]. However, different algae may show different physiological and biochemical responses to changing salinity conditions, and different species may also activate different antioxidant defense systems when exposed to extreme salinities [99,100]. Consequently, it is important to explore how different species react to a changing salinity environment, from both ecological and biotechnological/industrial perspectives, in regard to aquaculture candidate seaweed species. From our findings, consistently high nutrient levels, together with high solar radiation and salinity shock, may play an effective role in inducing the antioxidant compounds in specific Chlorophyta and Rhodophyta seaweeds. 

The ABTS assay was selected to evaluate antioxidant capacity because it is considered a fast and simple assay that provides a comprehensive view of the entire extract in both the lipophilic and hydrophilic medium [101]. Generally, the maximal antioxidant capacity of the studied seaweed was in line with other and similar red and green seaweed species from previous studies that had presented high antioxidant values, ranging up to 3.6 μg TE mg^−1^ DW [101,102,103].

Phenolic compounds are characterized as stress-induced compounds, involved in the chemical protection mechanisms against abiotic factors [53]. Polyphenol content was evaluated for *U. rigida* and revealed a similar trend to that of the antioxidant capacity, in which the highest phenolic content was observed under the high-salinity and the fish-integrated conditions. Similar to our results, Kumar et al. [87] also described a significant accumulation of polyphenols, particularly at a salinity of 45 ppt, following 6 days of culturing, suggesting that this could be a seaweed strategy to combat the salt stress. Antioxidant capacity can be associated with photoprotective compounds and secondary metabolites, such as phenols, proteins, protective pigments, and MAAs [30,35,103,104]. In the present study, antioxidant capacity demonstrated significant correlative relationships with polyphenols in *U. rigida*. A significant positive correlation was also observed for *H. musciformis* between its antioxidant capacity, SPF values, and its specific and total MAA levels, while in *G. confera*, the antioxidant capacity was positively correlated with phycobiliprotein levels (Supplementary S2.3). 

Seaweed-derived antioxidants, including polyphenols and other defensive compounds, are of special interest in the cosmetic, pharmaceutical, and nutrition fields. They already have important applications in a range of products, due to their bioactive properties that can protect the human body from free radicals and retard the progress of many chronic diseases, such as hypertension, heart diseases, diabetes and cancer [105,106,107]. Moreover, seaweed antioxidants offer a natural alternative to synthetic antioxidants, which may present potential toxicity and health risks [108]. 

### 3.4. SPF Manipulation 

Sun Protection Factor (SPF) is the universal indicator for the photoprotective capabilities of a product/substance against UV-B radiation [109]. The higher the SPF, the more effective the product is in preventing sunburn. This can be determined in vivo on human volunteers, by calculating the ratio of the least amount of ultraviolet energy required to produce a minimal erythemal dose on protected skin compared to unprotected skin [110]. SPF can also be determined in vitro, which is considered a faster, simpler, and less expensive method. There are several types of in vitro methods, with the more widely used approach being to evaluate the absorption characteristics of sunscreen agents based on spectrophotometric analysis of diluted solutions [111,112,113]. This can be used for preliminary purposes during production, in the analysis of the final product, and can provide important information before proceeding to the in vivo tests [114]. As has been further discussed in the current work, seaweeds may offer a potentially rich source of antioxidant and sunscreen compounds due their characteristic life history. These capabilities make them excellent candidates for utilization as ingredients for natural sunscreens [112,115,116]. In the current work, a SPF standardization for 1 mg seaweed DW in 1 mL aqueous solvent was used. The SPF found for *Ulva rigida*, *Gracilaria conferta*, and *Hypnea musciformis* was between 1 and 4, indicating their potential use for sunscreen applications [113]. Similar to the trends observed in the MAAs and their antioxidant capacity, the highest SPF values were observed under the fish-integrated conditions and high-salinity treatments. MAAs, phycobiliproteins, and phenols are also water-soluble molecules, and this might also have contributed to the high SPF values obtained. An especially important finding in the present work is that SPF differences of up to 270% could be identified between the different culture treatments. This illustrates that by controlling the seaweed cultivation conditions, it is possible to direct and increase their sunscreen and photoprotective properties, nurturing them specifically for this application.

## 4. Materials and Methods

### 4.1. Integrated Aquaculture System and Experimental Design

**Figure 6:**
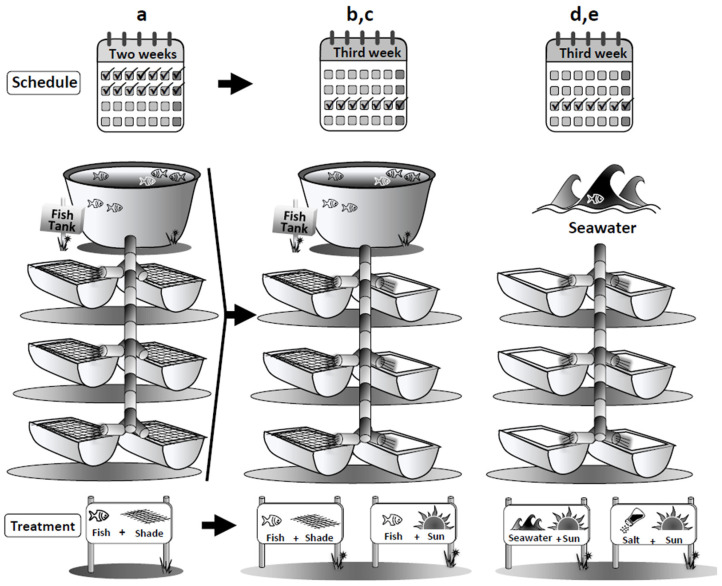
Layout of the experimental system: (**a**) integrated cultivation in the initial two weeks, (**b**) integrated cultivation in the third week, (**c**–**e**) third week environmental stresses.

The integrated mariculture setup used in the current study was designed and installed in a land-based experimental seaweed site at the Israel Oceanographic and Limnological Research center in Haifa, Israel, as described in our earlier work [23]. The integrated system had two components: (1) a culture tank stocked with gilthead sea bream fish (*Sparus aurata*), and (2) a series of seaweed cultures tanks (n = 6) alternatively stocked with one of three local macroalgae species, the green macroalga *Ulva rigida,* or one of two red macroalgae, *Gracilaria conferta* and *Hypnea musciformis*. Filtered seawater was first diverted to the fish tanks and then channeled through PVC pipes by gravity into the seaweed culture tanks. Control tanks that received only regular seawater were stationed parallel to the system. Initially, each seaweed species was cultivated in the system with the sea-bream fish effluents during two consecutive weeks. Following these two weeks, 100 g FW biomass was randomly collected from the system and distributed into 4 different culture treatment tanks which included a second control and 3 different abiotic stresses that run for one additional week (n=3 seaweed tanks for each treatment), as depicted in Figure 6 and detailed as follows: 

**Control, Seawater + Shade:** shaded tanks receiving about 50% of full sunlight and supplied with regular seawater.

**Treatment 1, Fish + Shade** (run for the first initial two weeks, and at the third week to represent a second control): shaded tanks receiving about 50% of full sunlight and supplied with fishpond effluents.

**Treatment 2, Fish + Sun:** unshaded tanks receiving 100% of full sunlight, supplied with fishpond effluents intended to inflict sunlight stress [54].

**Treatment 3, Seawater + Sun:** unshaded tanks receiving 100% of full sunlight supplied with regular seawater to create a nutrient-limited environment combined with a sunlight stress [52,54].

**Treatment 4, Salt:** high salinity cultivation of about 45–55 ppt [87].

### 4.2. Chemical Composition of Seaweed Tissues: Sample Preparation 

At the start and end of each stage of the experiments, seaweed thalli from each culture tank were carefully washed with tap water to discard salt, debris, and epiphytes, and finally centrifuged with a kitchen spinner to remove excess water. Samples were then freeze-dried using a lyophilizer (Christ, Alpha 1-2 LD plus, Osterode am Harz, Germany), grounded to a fine homogenized powder and stored at −20 °C, prior to further chemical analyses. Triplicates from each of the treatments and from each tank were taken for the different chemical analyses.

#### 4.2.1. Analysis of Mycosporine-like Amino Acids (MAAs)

MAAs were assayed according to Korbee-Peinado et al. [61], where 50 mg of dried seaweed was incubated in 20% methanol (1 mL) in a water-bath at 45 °C for 2 h. Then, 700 μL of the supernatant were taken and evaporated under vacuum at 45 °C (SpeedVac SPD210 Vacuum Concentrator, Thermo scientific, Waltham, MA, USA). Dried extracts were redissolved in 700 μL 100% methanol and vortexed for 30 s. After passing through a 0.2-μm membrane filter, samples were analyzed with an Agilent UHPLC system (1260 Agilent InfinityLab Series, Santa Clara, CA, USA). Identification of MAAs was performed by comparison of the absorption spectra and retention times with characterized co-standard, *Pyropia leucosticta*, characterized by the MAAs porphyra-334, shinorine, palythine, asterina-330 and palythinol, that were previously identified by mass spectrometry in Chaves-Peña et al. [117]. Quantification was carried out by using published extinction coefficients [118,119,120,121,122]. Results were expressed as mg g^−1^ DW.

#### 4.2.2. Pigment Extraction and Evaluation

Pigment content (mg g^−1^ DW) was evaluated using the method described by Osorio et al. [80], with minor alternations. Chlorophylls were extracted with 90% acetone, and phycobiliproteins (phycoerythrin and phycocyanin) using 0.1 M phosphate buffer (pH 6.8). Extractions were carried out in triplicate by adding 100 mg of dried seaweed to 20 mL of each solvent. Following the first extraction, samples were placed in an ultrasonic bath for 30 min [123]. Samples were than vortexed and remained overnight in darkness at 25 °C. Finally, extracts were centrifuged at 4000 rpm for 20 min, and supernatants were taken for analyses. Absorbance was read via a Agilent Cary 60 UV-Vis Spectrophotometer (Santa Clara, CA, USA), with quartz cuvettes, and concentrations were calculated using the equations described below [80,124]:

Chl *a* (µg mL^−1^) = −0.3319 × (A_630_ − A_750_) − 1.7485 × (A_647_ − A_750_) + 11.9442 × (A_664_ − A_750_) − 1.4306 × (A_691_ − A_750_) (±0.0020)

Chl *b* (µg mL^−1^) = −1.2825 × (A_630_ − A_750_) + 19.8839 × (A_647_ − A_750_) − 4.8860 × (A_664_ − A_750_) − 2.3416 × (A_691_ − A_750_) (±0.0076)

Chl *d* (µg mL^−1^) = −0.5881 × (A_630_ − A_750_) + 0.0902 × (A_647_ − A_750_) − 0.1564 × (A_664_ − A_750_) + 11.0473 × (A_691_ − A_750_) (±0.0030)

Total Chl (µg mL^−1^) = 21.3877 × (A_630_ − A_750_) + 10.3739 × (A_647_ − A_750_) + 5.3805 × (A_664_ − A_750_) + 5.5309 × (A_691_ − A_750_) (±0.0056)

$$\mathrm{Phycoerythrin}\;(µg\;{\mathrm{mL}}^{-1})=\frac{A_{565-}A_{750}}{2.41\times 10^{6}}\times 240,000\times 10^{3}$$

$$\mathrm{Phycocyanin}\;(µg\;{\mathrm{mL}}^{-1})=\frac{A_{618-}A_{750}}{1.90\times 10^{6}}\times 264,000\times 10^{3}$$

#### 4.2.3. Determination of Antioxidant Activity

The seaweed antioxidant activity was evaluated using the ABTS method [125]. Seaweed samples were first extracted by adding 1 mL of phosphate buffer (0.1 M, pH = 6.5) to 20 mg of dry seaweed powder. The samples were vortexed and remained overnight in darkness at 4 °C. Later, extracts were centrifuged, and supernatants were collected for analyses. ABTS reagent was prepared in sodium phosphate buffer (0.1 M, pH 6.5), using ABTS (2,2-azino-bis (3-ethylbenzothiazoline-6-sulphonic acid, 7 mM) and potassium persulfate (K2S2O8, 2.45 mM). The reagent was incubated in darkness at room temperature for 12–16 h, allowing complete formation of the radical. The assay reaction was performed by adding 950 μL of diluted ABTS reagent and 50 μL of each seaweed extract. The samples were agitated, and absorbance was recorded by a UV–visible spectrophotometer (UV-2700i Shimadzu, Duisburg, Germany) at 727 nm after 8 min of incubation. The blank was phosphate buffer. The antioxidant activity was calculated using the following formula:

AA% = [(ODi − ODf)/ODi] × 100

Quantification of antioxidant compounds was determined using a standard curve with different Trolox (6-hydroxy-2,5,7,8-tetramethylchroman-2-carboxylic acid) concentrations. The results were expressed as μg of Trolox Equivalents (TE) per mg of seaweed dry weight (μg mg^−1^ DW).

#### 4.2.4. Determination of Phenolic Compounds

Quantification of phenolic compounds was performed according to the Folin–Ciocalteu method [126], with some modifications. Seaweed samples were first extracted as described above for the antioxidant activity. The reaction was performed by adding 100 μL of each seaweed extract to 700 μL of distilled water, 50 μL of the Folin–Ciocalteu reagent, and, finally, 150 μL of 20% anhydrous sodium carbonate (Na_2_CO_3_). The solution was vortexed and incubated at 4 °C in darkness for 2 h. Absorbance was measured at 760 nm using a UV–visible spectrophotometer (UV-2700i Shimadzu, Duisburg, Germany). The blank included all reagents, and the crude extract was replaced by distilled water. Phenolic content was evaluated by constructing a standard curve using different phloroglucinol concentrations. Results were expressed as μg of phloroglucinol equivalent (PE) per mg of seaweed dry weight (μg mg^−1^ DW).

#### 4.2.5. Sun Protection Factor (SPF) Evaluation

In vitro SPF values were determined according to the Mansur spectrophotometric method [127]. Prior to analysis, 3 different types of solvents, i.e., ethanol, ethyl acetate, and double-distilled water (DDW), previously used in similar studies [128,129], were tested to evaluate which generate the best value of SPF performance. DDW was chosen after obtaining the highest values. Extraction was performed by adding 25 mL of DDW to 100 mg of dry seaweed powder. The samples were vortexed, placed in an ultrasonic bath for 30 min, and then remained overnight in the darkness at 25 °C. Finally, extracts were centrifuged, and supernatants were used for analyses. Absorbance was measured between 290 and 320 nm with a 1-cm quartz cell at 5-nm intervals using Agilent Cary 60 UV-Vis Spectrophotometer (Santa Clara, CA, USA). DDW was used as the blank. The SPF values were standardized for a final concentration of 1 mg seaweed DW in 1 mL of solvent (1 mg mL^−1^), and was calculated by using the equation derived by Mansur et al. [127] and Malsawmtluangi et al. [113]:

$$\mathrm{SPF}\;=\mathrm{CF}\times \sum_{290}^{320}\;\mathrm{EE}\left(\lambda \right)\times I\;\left(\lambda \right)\times \mathrm{Abs}\;\left(\lambda \right)\;\;$$

where CF = correction factor (=10), EE (λ) = erythemaogenic effect of radiation with wavelength λ, and Abs (λ) = spectrophotometric absorbance values at wavelength λ. The values of EE(λ) × I(λ) are constants and were determined by Sayre et al. [111]. 

### 4.3. Statistical Analysis

Statistical analyses were performed using the R statistic program, version 4.0.2, Vienna, Austria. One-way ANOVA (*a* = 0.05) was used to compare parameters between experiments. Tukey’s HSD test was used for post hoc pairwise comparisons. Data were tested for normality (Shapiro–Wilks test) and homogeneity of variance (Levene test). When ANOVA assumptions were not met, a permutation ANOVA test of 5000 repetitions was used and a Games–Howell test was applied for post hoc comparison. Data in tables and figures are expressed as mean ± SD.

## 5. Conclusions and Future Perspectives

Seaweeds account for almost 3000 different natural products, representing about 20% of the entire chemistry of the marine realm. Research on marine natural products has proliferated since the 1960s, when seaweeds were at the center of new discoveries. Since then, attention in the field has shifted to microalgae [130]. However, microalgae production for mass industrial use still has several drawbacks, and their bio-refinery cost may be less economically viable compared to that of seaweed cultivation [29,131,132]. The seaweeds’ attributes of high yields and growth, high bioremediation capabilities, and chemical richness, make them excellent future candidates for the mass cultivation of sustainable and functional high-value biomass.

*Ulva*, *Gracilaria* and *Hypnea* are intertidal seaweed genera that possess chemical defenses and protection mechanisms, including the ability to increase secondary metabolites, such as MAAs and phenolic compounds, and alter their pigment levels, thereby allowing them to thrive under changing environmental conditions. 

Our intention in the current work was to artificially increase the seaweeds’ metabolism in order to enhance specific and valuable antioxidant and photoprotective biomaterials. We were able to devise a practical approach based on a two-step/phase cultivation scheme, in which the seaweeds were initially grown alongside fish effluents, and subsequently exposed to various abiotic conditions (stressors). The two-phase cultivation method was inspired by work performed in β-carotene production in *Dunaliella* [133]. Although this approach is customarily applied to microalgae cultivation [134], it is new and yet to be routinely established for seaweed cultivation.

Table 3 depicts how each of the seaweed compounds/attributes can be manipulated and enhanced based on our study’s findings, where it can be practically adopted by the seaweed industry. Possibly, other unique attributes of different seaweed species could be enhanced. By using our cultivation approach, additional seaweed-derived bioactive compounds, such anti-biotic, anti-viral, anti-inflammatory, anti-diabetic, and anti-cancer substances, could potentially be manipulated and their concentrations increased. 

The IMTA cultivation provided the seaweeds with ideal growing conditions, accelerating their biosynthesis towards high yields and N compound production, while also providing them with the building blocks necessary to support their chemical defense mechanisms in times of stress. Additionally, the practice of seaweed IMTA offers an important sustainable advantage, since the seaweeds assimilate the inorganic nutrients from the water, thus minimizing the risk of coastal eutrophication [135]. 

The findings from this study confirm that under the high nutrient concentrations provided by the IMTA system, together with sufficient solar radiation, and/or followed by high salinity shock, the total content of MAAs, pigments, and phenolic compounds, as well as their antioxidant capacity and SPF, can be stimulated significantly in the seaweeds by several hundred percent. The enhanced seaweed biomass can be used as a quality raw material for healthy foods and additives, for health-promoting pharma and cosmetic products, and for further bio-refinery and extraction processes.

## Figures and Tables

**Table 1:** Biological parameters of the seaweed species examined in the study. Evaluation was preformed using dry weight (DW). Data refer to maximal values attained among the different treatments. (-) no available data.

Parameter	*Ulva rigida*	*Gracilaria* *conferta*	*Hypnea* *musciformis*
Antioxidant activity (µg TE mg^−1^)	3.5 ± 0.15	2.3 ± 0.25	4.5 ± 0.31
Phenolic compounds (µg PE mg^−1^)	4.7 ± 0.3	-	
SPF (mg mL^−1^)	3.82 ± 0.16	1.82 ± 0.1	3.12 ± 0.12
Total MAAs (mg g^−1^)	-	1.8 ± 0.7	3.3 ± 0.5
Palythine (mg g^−1^)	-	0.2 ± 0.06	0.8 ± 0.16
Asterina-330 (mg g^−1^)	-	0.02 ± 0.02	0.06 ± 0.01
Palythinol (mg g^−1^)	-	1.5 ± 0.6	2.05 ± 0.7
Shinorine (mg g^−1^)	-	0.3 ± 0.1	1 ± 0.2
Porphyra-334 (mg g^−1^)	-	0.1 ± 0.02	0.05 ± 0.01
Chlorophyll *a* (mg g^−1^)	3.9 ± 0.2	0.5 ± 0.1	1.2 ± 0.2
Phycoerythrin (mg g^−1^)	-	4.1 ± 1.1	7.5 ± 1.8
Phycocyanin (mg g^−1^)	-	1.6 ± 0.5	6.6 ± 1.2

**Table 2:** Effect of different culture conditions on the pigment levels in the studied seaweeds (mean ± S.D, n = 3).

Species	Culture Condition/Treatment	Chlorophyll *a* (mg g^−1^ DW)	Chlorophyll *b* (mg g^−1^ DW)	Chlorophyll *d* (mg g^−1^ DW)	Total Chlorophylls (mg g^−1^ DW)	Phycoerythrin (mg g^−1^ DW)	Phycocyanin (mg g^−1^ DW)
*Ulva rigida*	Control	0.71 ± 0.05	0.36 ± 0.02	-	1.06 ± 0.06	-	-
	Fish + Shade (initial two weeks) *	3.89 ± 0.17	2.08 ± 0.09 *	-	5.9 ± 0.24 *	-	-
	Fish + shade	1.66 ± 0.12	1.06 ± 0.06	-	2.61 ± 0.18	-	-
	Fish + sun	1.80 ± 0.28	1.12 ± 0.18	-	2.78 ± 0.43	-	-
	Seawater + sun	0.31 ± 0.11	0.18 ± 0.07	-	0.47 ± 0.17	-	-
	High salinity	0.38 ± 0.04	0.23 ± 0.03	-	0.58 ± 0.07	-	-
*Gracilaria conferta*	Control	0.12 ± 0.01	-	0.002 ± 0.001	0.12 ± 0.01	0.57 ± 0.05	0.31 ± 0.03
	Fish + Shade (initial two weeks) *	0.47 ± 0.09	-	0.01 ± 0.002	0.49 ± 0.08 *	4.14 ± 1.16 *	1.64 ± 0.48 *
	Fish + shade	0.34 ± 0.03	-	0.01 ± 0.002	0.38 ± 0.04	2.5 ± 0.25	1.06 ± 0.05
	Fish + sun	0.38 ± 0.08	-	0.005 ± 0.001	0.45 ± 0.11	2.03 ± 0.68	0.95 ± 0.29
	Seawater + sun	0.14 ± 0.02	-	0.004 ± 0.002	0.16 ± 0.03	0.64 ± 0.08	0.35 ± 0.03
	High salinity	0.09 ± 0.01	-	0.01 ± 0.0004	0.11 ± 0.01	0.36 ± 0.04	0.2 ± 0.02
*Hypnea musciformis*	Control	0.34 ± 0.02	-	0.01 ± 0.001	0.39 ± 0.02	1.19 ± 0.08	0.6 ± 0.06
	Fish + Shade (initial two weeks) *	1.18 ± 0.19	-	0.05 ± 0.01	1.32 ± 0.2 *	7.49 ± 1.77 *	6.63 ± 1.25 *
	Fish + shade	0.68 ± 0.18	-	0.04 ± 0.01	0.82 ± 0.21	3.88 ± 0.98	3.47 ± 1.03
	Fish + sun	0.62 ± 0.22	-	0.03 ± 0.01	0.75 ± 0.25	2.89 ± 1.12	2.5 ± 1.08
	Seawater + sun	0.31 ± 0.06	-	0.01 ± 0.002	0.36 ± 0.07	1.06 ± 0.32	0.97 ± 0.29
	High salinity	0.35 ± 0.02	-	0.01 ± 0.001	0.4 ± 0.02	1.27 ± 0.18	1.28 ± 0.1

* treatments with the highest significant values. (-) no available data.

**Table 3:** Seaweeds’ general response to the different treatments. Higher (green, 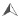), considerably higher (green, 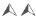), lower (red, 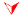), no major effect (—).

Treatment	Control Seawater + Shade	Fish Effluent + Shade	Fish Effluent + Shade	Fish Effluent + Sun	Seawater + Sun Shock	Salt Shock
Time		Initial two weeks	Third week			
Total MAAs	—	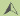	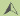	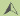	—	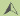
Chlorophylls	—	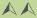	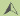	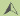	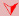	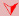
Phycobiliproteins	—	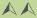	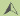	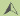	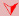	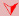
Antioxidant activity	—	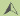	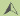	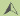	—	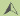
Phenolic compounds	—	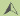	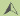	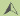	—	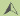
SPF	—	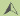	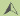	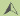	—	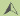

## Data Availability

The data presented in this study are available in the text and in Supplementary S1.

## Supplementary Materials

The following supporting information can be downloaded at: https://www.mdpi.com/article/10.3390/md20120767/s1. Supplementary S1: Statistical analysis for different compounds and parameters examined in the study: MAAs (mg g^−1^ DW), Antioxidant activity (µg TE mg^−1^ DW), Polyphenols (μg PE m g^−1^ DW), SPF, and pigment concentration (mg g^−1^ DW) of *Ulva rigida*, *Gracilaria conferta*, and *Hypnea musciformis*. When sample size (N) was not large enough for the Games–Howell post hoc test, Tukey’s HSD was used instead for pairwise comparisons. System refers to Fish + Shade: integrated cultivation with the fish during the initial two weeks. Supplementary S2.1: Concentrations of dissolved nutrients, including total ammonia nitrogen TAN (NH_4_ and NH_3_), nitrate (NO_3_), and phosphate (PO_4_), monitored at the integrated seaweed tanks following the fish culture, throughout the experimental period (average values) for each seaweed species. Seawater refers to the ambient seawater concentrations. Inlet refers to the water coming into the seaweed cultivation tanks following the fish culture (rich with nutrients), and outlet refers to the water flowing out of the seaweed cultivation tanks (after being biofiltered). Further values and methods used were described in our earlier work [23]. Supplementary S2.2: The individual MAA contents (mg g^−1^) for *Gracilaria conferta* and *Hypnea musciformis*, cultivated under the different environmental conditions. Evaluation was preformed using dry weight (DW). Supplementary S2.3: Pearson coefficient (r) between the antioxidant activity and the different compounds/attributes evaluated in the present study for the three species: *Ulva rigida*, *Gracilaria conferta*, and *Hypnea musciformis*. Green: positive correlation. (-) no available data. ** *p* < 0.01, * *p* < 0.05.
