# Supplementary material for: Enhancing Bioproducts in Seaweeds via Sustainable Aquaculture: Antioxidant and Sun-Protection Compounds
Source: Mar Drugs. 2022 Dec 7;20(12):767. doi: 10.3390/md20120767 (PMC9787370; doi:10.3390/md20120767)
Supplement: Supplementary file 1 [file marinedrugs-20-00767-s001.zip › marinedrugs-2016616-Supplementary S2.pdf]

Supplementary S2.1

Concentrations of dissolved nutrients, including total ammonia nitrogen TAN (NH_4 and NH_3), nitrate (NO_3) and phosphate (PO_4), monitored at the integrated seaweed tanks following the fish culture, throughout the experimental period (average values) for each seaweed species. Seawater, refers to the ambient seawater concentrations. Inlet, refers to the water coming into the seaweed cultivation tanks following the fish culture (rich with nutrients), and outlet, refers to the water flowing out of the seaweed cultivation tanks (after being biofiltered). Further values and methods used were described in our earlier work [23].

	Hypnea musciformis			***Ulva rigida***			***Gracilaria conferta***		
	**Seawater**	**Inlet**	**Outlet**	**Seawater**	**Inlet**	**Outlet**	**Seawater**	**Inlet**	**Outlet**
**TAN (mg L^{-1})**	0.003±0.003	0.53±0.03	0.23±0.06	0.006±0.001	0.36±0.1	0.1±0.08	0.009±0.006	0.47±0.07	0.25±0.07
**NO_3 (mg L^{-1})**	0.05±0.01	0.06±0.0003	0.03±0.01	0.05±0.01	0.05±0.2	0.03±0.01	0.05±0.002	0.07±0.01	0.05±0.01
**PO_4 (mg L^{-1})**	0.001±0.001	0.09±0.004	0.06±0.01	0.002±0.001	0.05±0.04	0.03±0.02	0.003±0.002	0.11±0.01	0.07±0.02
		**Inlet**			**Inlet**			**Inlet**	
**TAN (g m^{-2} d^{-1})**	0.03±0.03	5.07±0.33		0.07±0.01	3.72±1.09		0.1±0.06	4.89±0.75	
**PO_4 (g m^{-2} d^{-1})**	0.01±0.01	0.86±0.04		0.03±0.01	0.53±0.39		0.03±0.02	1.14±0.15	

Supplementary S2.2

The MAA contents (mg g^{-1}) for *Gracilaria conferta* and *Hypnea musciformis*, cultivated under the different environmental conditions. Evaluation was preformed using dry weight (DW).

Gracilaria conferta

	Culture condition	Palythine (mg g^{-1})	Asterina-330 (mg g^{-1})	Palythinol (mg $^{g-1}$)	Shinorine (mg g^{-1})	Porphyra-334 (mg g^{-1})
Initial two weeks	Control	0.01	0.00	0.56	0.08	0.10
	Fish + Shade	0.02	0.00	0.70	0.08	0.09
Third Week	Fish + Shade	0.01	0.00	0.85	0.12	0.09
	Fish + Sun	0.05	0.00	0.93	0.20	0.04
	Seawater + Sun	0.05	0.01	1.45	0.30	0.01
	Salt	0.21	0.02	0.74	0.13	0.00

Hypnea musciformis

	Culture condition	Palythine (mg g^{-1})	Asterina-330 (mg g^{-1})	Palythinol (mg g^{-1})	Shinorine (mg g^{-1})	Porphyra-334 (mg g^{-1})
Initial two weeks	Control	0.28	0.02	0.63	0.47	0.00
	Fish + Shade	0.23	0.01	2.05	0.82	0.03
Third Week	Fish + Shade	0.29	0.02	1.40	0.98	0.02
	Fish + Sun	0.49	0.04	1.69	1.00	0.03
	Seawater + Sun	0.44	0.02	0.71	0.38	0.00
	Salt	0.81	0.06	1.67	0.76	0.05

Supplementary S2.3

Pearson coefficient (r) between the antioxidant activity and the different compounds/attributes evaluated in the present study for the three species: *Ulva rigida*, *Gracilaria conferta*, *Hypnea musciformis*. Green: Positive correlation. (-) no available data. ** p<0.01, *p<0.05.

	Compound										
species	Palythine	Asterina 330	Palythinol	Shinorine	Porphyra 334	Total MAAs	Phycoerythrin	Phycocyanin	Total chlorophylls	polyphenols	SPF
Ulva rigida	-	-	-	-	-	-	-	-	-0.3223	**0.7224 ****	0.066
Gracilaria conferta	0.4316	0.3146	-0.2547	-0.3616	-0.07	-0.2232	**0.527 ***	**0.5 ***	0.435	-	0.2894
Hypnea musciformis	0.4136	**0.5224 ***	**0.6284 ****	**0.6724 ****	**0.5924 ****	**0.7332 ****	0.3435	0.4147	0.416	-	**0.8794 ****
